# The New Independence; Made in America; Patriotism in the Medical Profession

**Published:** 1915-06

**Authors:** G. Henri Bogart

**Affiliations:** Paris, Illinois


### The New Independence; Made in America; Patriotism in the Medical Profession.

BY DR. G. HENRI BOGART, PARIS, ILLINOIS.

When the great war first commenced the medical man wondered where he was to obtain the customary drugs. Now the restrictions of commerce have put the hobble on the other foot, and the hemmed-in belligerents, and lots of other folks, are wondering about the food supply. The whole world has been crying for the moon, turning to the distance for the glamour, passing up the "something just as good" or better at home.

The Martians may be supposed to gaze down at their evening star, our earth, in its wondrous purity of white beauty, unthinking of the bottomless, spring-thawed roads of Illinois, or the discoveries of the vice commissions and other drawbacks; blotches on its silvery sheen. That star is our earth.

I have driven through the woodlands of Indiana when the hillsides glowed with acres of the purple splendor of the digitalis. The farmer cursed it, also the stagger weed that is poisoning his cattle, or compelled him to forego the use of the lush pasturage. Then I stopped at the drug store to purchase digitalis "Made in Germany"! I have seen the village physician paying a laborer to grub the "wandering milkweed" from his pasture lot, and at the same time prescribing the alkeloid from those same laboratories across the sea, while his father had learned the virtues of pleurisy root from the older botanic school. No, I do not mean that we shall go back, and with shears and shovel gather our sources for infusion and decoction.

America has splendid laboratories with well won reputation for alkeloids, specific tinctures and extracts second to none. We have unlimited fields of crude material, the plants and minerals in abundance; either neglected or harvested for shipment to the laboratories across the ocean, on whom we have come to depend.

We prate about our independence, and then depend, slavishly, upon those others, neglecting our own manufacturers. We cannot expect our own chemists to flourish unless we support them, for we read "that which grows by that it doth feed upon."

I have trellises of roses about my home, vines that yield wealth of perfumed beauty that would not be did I not furnish the support and loving care for their growing.

As a duty, as pure patriotism, we should declare a new era of independence and we need not wait until the Fourth of July either, rather "Act, act in the living present."

We need no Congress, no Liberty Hall; we need just you, and you, and you, all pulling together for the sustenance of home remedies.

I have been sometimes amused, sometimes disgusted at the prominence given "Twilight Sleep," with all the twaddle and rigamarole of the lay press, exploiting that something new that drew the Greeks about St. Paul on Mars Hill. Right here in America we have had for years a more dependable preparation in tablet form on the market, permanently dispensed, and with abundant free literature on the subject. Nothing has been said about it only in a business way to the profession; you could not have driven a newspaper or a magazine reporter to have peeped through the portals of the possibilities of this "Made in America" release from the pangs and terrors of childbirth, for the story would have been commercial, lacking the twang and novelty of importation. It would have violated the ethics of publication by advertising something as a news feature. This publication, with gratis literature, has been before the American public, meeting silence, however, while its more clumsy foreign substitute was boomed as a sensation, a scoop.

There is the axiom of supply and demand that answers the question as to the ability of our chemists and pharmacists to supply all the skill and products we need.

We annually spend many millions on scientific education, and then calmly lose our eyes and leave the men and women whom we have trained to potter and piddle, for want of a demand for their services that shall sharpen their faculties and reward their efforts, throttling their ambitions instead of spurring them on.

Here is one example of what incentive will do:

Edison was using tons of carbolic acid each week when the war stopped his supply. He had been told that our coals would not furnish it. He did not shut down his works; he investigated the making a synthetic product,—got busy. Within a month he was making a superior product, cheaper than the foreign chemist had produced it.

Incidentally a report comes from the Bureau of Mines of the Interior Department that is as revolutionary as illustrative. Walter F. Rittman, chemical engineer, while experimenting with crude petroleum as a possible source of tolulol and benzol as a practicable basis for the coal tar dyestuffs not only succeeded, but as part of the process discovered that he could get more than twice the amount of gasoline from a given quantity of petroleum than ever before. Both products were better standardized than under the old methods, and manufactured cheaper and with less danger.

The effects of the by-product discovery (more and better gasoline) is of incalculable monetary value. It is not in furnishing power to the two and a half millions of motor vehicles in America (enormous as this is, now that it is solving the problem of the street railway extortions), that gasoline is important. The gas engine in the factory, shop, in home and office, for irrigation and navigation or as an agricultural tractor (though not so apparent), is really used to a greater extent than the motor vehicle. These discoveries will be patented to make them free for the whole American people, and to remove them from the control of any trust or monopoly. They are only another example of a demand made strong enough to win the supply.

America can furnish as good chemicals, if not better, than the foreigner, if the rank and file will support the demand. Our chemists and pharmacists are able, willing and anxious to furnish for our utmost needs if allowed the opportunity. If you are not satisfied with the reactions of a remedy, if you need something that it lacks, tell your manufacturer; write him candidly; take him into your confidence; he is more than willing to co-operate.

If you know what you want he will be more than willing to meet you half way; and more, to solve your difficulties.

Be patriotic in the highest sense, that of the upbuilding of your own country, which is better than hurting and destroying your neighbor nation.

Make yourself one of the signers of the new Declaration of Independence, and do it now, and having done it, keep on doing it.

